# Serial dependencies and overt attention shifts

**DOI:** 10.1167/jov.25.14.12

**Published:** 2025-12-17

**Authors:** Sandra Tyralla, Eckart Zimmermann

**Affiliations:** 1 Department of Experimental Psychology, Heinrich Heine University Düsseldorf, Düsseldorf, Germany

**Keywords:** saccadic adaptation, serial dependency, orientation judgement, overt attention

## Abstract

When visual input is uncertain, visual perception is biased toward the stimulation from the recent past. We can attend to stimuli either endogenously based on an internal decision or exogenously, triggered by an external event. Here, we wondered whether serial dependencies are selective for the attentional mode which we draw to stimuli. We studied overt attention shifts: saccades and recorded either motor error correction or visual orientation judgments. In Experiment 1, we assessed sensorimotor serial dependencies, focusing on how the postsaccadic error influences subsequent saccade amplitudes. In Experiment 2, we evaluated visual serial dependencies by measuring orientation judgments, contingent on the type of saccade performed. In separate sessions, participants performed either only voluntary saccades or only delayed saccades, or both saccade types alternated within a session. Our results revealed that sensorimotor serial dependencies were selective for the saccade type performed. When voluntary saccades had been performed in the preceding trial, serial dependencies were much stronger in the current trial if voluntary instead of delayed saccades were executed. In contrast, visual serial dependencies were not influenced by the type of saccade performed. Our findings reveal that shifts in exogenous and endogenous attention differentially impact sensorimotor serial dependencies, but visual serial dependencies remain unaffected.

## Introduction

Perceptual processing must constantly compensate for noisy sensory input. One method to do so has been discovered in the last decade and consists of a reliance of current stimulus interpretation on the recent past. Fischer and Whitney (2014) reported that, when observers had to report the orientation of stimuli presented in the visual periphery, their estimates were biased by the orientation of the stimulus presented in the previous trial. In other words, interpretation of noisy sensory input is serially dependent on similar stimulation from the recent past. Most serial dependencies that have been reported are positive, such that the current stimulus appears a bit more similar to the previous one (Liberman, Zhang, & Whitney, 2016; Taubert, Van Der Burg, & Alais, 2016; Tyralla & Zimmermann, 2024). Since then, serial dependencies have been found in various domains such as visual orientation (Fischer & Whitney, 2014; Fritsche & de Lange, 2019; Rafiei, Hansmann-Roth, Whitney, Kristjansson, & Chetverikov, 2021), shape (Collins, 2022; Manassi et al., 2021; Manassi, Kristjánsson, & Whitney, 2019), color (Bays, Catalao, & Husain, 2009; Barbosa & Compte, 2020), numerosity (Cicchini, Anobile, & Burr, 2014; Fornaciai & Park, 2018), visual stability (Manassi & Whitney, 2022), or saccadic eye movements (Cont & Zimmermann, 2021). Serial dependencies are believed to be the signature of a mechanism that stabilizes perception (Cicchini, Mikellidou, & Burr, 2024; Manassi & Whitney, 2024). If our environment is successfully integrated into a stable perception, these authors argue, then object features will result in smooth and continuous perception. We have recently reported sensorimotor serial dependencies that exist between motor errors and perceptual estimates (Cont & Zimmermann, 2021). When subjects were required to perform a saccade, an artificial error was created by displacing the saccade target during movement execution. The amplitude of the immediately following saccade was shaped by this postsaccadic error. If subjects were asked to visually localize a target in space after having experienced a postsaccadic error in the previous trial, their estimate in the current trial was likewise biased by the preceding one.

However, serial dependencies operate within certain limits. Perception would be tremendously impaired if every object biases every other. Serial dependencies are temporally and spatially tuned such that only objects close in space and time affect each other (Fischer & Whitney, 2014; Manassi, Liberman, Kosovicheva, Zhang, & Whitney, 2018). Several studies have suggested that visual serial dependencies affect the current stimulus only if the previously encountered stimulus has been attended (Bae & Luck, 2020; Fischer & Whitney, 2014; Fornaciai & Park, 2018; Liberman et al., 2016; Rafiei et al., 2021). Fischer and Whitney (2014) presented eight oriented targets and a cue indicating which of the targets was to be judged. They found significant serial dependencies only if the cue validity was 100%. In another approach, Bae and Luck (2020) demonstrated that not only the target but also its specific feature had to be attended to produce serial dependencies. They found serial dependencies for motion only if subjects had to report the direction of motion but not if they reported the color of the same stimulus in the previous trial. However, other studies have not found any effects of attention on serial dependencies (Fornaciai & Park, 2018; Goettker & Stewart, 2022). In a meta-analysis, Manassi, Murai, and Whitney (2023) found that devoting fewer attentional resources to the previous stimulus resulted in reduced serial dependencies.

Allocation of attention can be divided into two modes (Chica, Bartolomeo, & Lupiáñez, 2013). Endogenous attention is drawn deliberately to objects of interest. Exogenous attention is triggered by a sudden event in the external world that leads to an automatic attention shift to its location (Carrasco & Barbot, 2015). Deployment of exogenous attention is transient, builds up for ∼100 to 120 ms, and decays quickly. Endogenous attention, in contrast, takes longer to build up (∼300 ms) and can be upheld as demanded (Godijn & Pratt, 2002). Attention shifts improve the processing of visual contrast and spatial resolution. The effects of exogeneous and endogenous attention shifts differ (Barbot & Carrasco, 2017; Yeshurun & Carrasco, 1998). For spatial resolution, exogenous attention improves spatial resolution in the visual periphery at the cost of central information. Endogenous attention can improve perception simultaneously at peripheral and central locations. A recent study investigated the impact of exogenous and endogenous covert attention shifts on the sensory tuning of orientation. Both modulate sensory tuning by changing its gain, with exogenous attention having stronger orientation gain enhancement (Fernández, Okun, & Carrasco, 2022).

Attention can also be divided in the way it is drawn to objects of interest. We can attend either covertly to objects or events while keeping our eyes still or overtly by performing an eye movement. Although the differences between exogenous and endogenous attention have mostly been studied in covert attention shifts, they can also be observed in overt attention shifts. When a sudden visual event appears, we perform a delayed saccade to it automatically. However, we can also execute voluntary saccades, moving the eye to a target selected by an internal decision. Shortly before a saccade is executed, attention shifts mandatorily to the saccade target location (Deubel & Schneider, 1996; Hoffman & Subramaniam, 1995; Kowler, Anderson, Dosher, & Blaser, 1995; Shepherd, Findlay, & Hockey, 1986; Van der Stigchel & Theeuwes, 2007). Overt and covert attention shifts are assumed to be coupled (Awh, Armstrong, & Moore, 2006; Corbetta, 1998; Smith & Schenk, 2012).

In the current study we asked whether exogenous and endogenous overt attention shifts would differentially affect serial dependencies. If the separate effects of exogenous and endogenous covert attention shifts on neural orientation tuning take place likewise in overt attention shifts, then the magnitude of serial dependencies might vary. For sensorimotor serial dependencies, results from saccade adaptation suggest differences for overt exogenous and endogenous attention shifts. In saccade adaptation, the amplitude adjustment in response to the previous postsaccadic error increases across trials until it reaches a steady-state level (Hopp & Fuchs 2004; McLaughlin, 1967; Pélisson, Alahyane, Panouillères, & Tilikete, 2010). Different saccade types have been tested in saccade adaptation experiments. The adaptation transfer between delayed and voluntary saccade types has been tested (Alahyane et al., 2007; Collins & Doré-Mazars, 2006; Deubel, 1995; Erkelens & Hulleman, 1993). These studies revealed that the adaptation of voluntary saccades substantially transfers to delayed saccades, whereas delayed saccade adaptation does not transfer to the same extent to voluntary saccades. It is yet unclear why the transfer is different. On the one hand, amplitude adjustments might occur at programming stages that only partly overlap for both saccade types. On the other hand, the presentation duration of the saccade targets that differ for delayed and voluntary saccades might produce the asymmetric adaptation transfer. Consistent with this view, studies have shown that adaptation of voluntary saccades affects the localization of stationary and flashed visual targets, whereas the adaptation of delayed saccades affects the localization of flashed targets only (Schnier, Zimmermann, & Lappe, 2010; Zimmermann & Lappe, 2009).

## Methods

### Participants

Twenty-three subjects (mean age ± *SD*, 21.13 ± 2.49 years; 19 women) participated in Experiment 1, and 23 subjects (mean age ± *SD,* 22.71 ± 5.22 years; 18 women) participated in Experiment 2. Participants were German native speakers, reported normal vision or wore lenses during the experiment, and indicated no psychiatric or neurological diseases. Participants were recruited at the Heinrich Heine University Düsseldorf. They either received course credits or 10 euros per hour for participation.

### Setup

Stimuli were presented on a 12.9-inch Diamond Pro 2070 cathode ray tube (CRT) screen (Mitsubishi, Tokyo, Japan) with 800 × 600-pixel resolution and a refresh rate of 120 Hz. In both experiments, subjects were placed 57 cm away from the screen in a dark room. A transparent foil reduced the luminance of the monitor by 2 log units and prevented the visibility of the monitor borders. We used a homogeneous gray background (0.09 cd·m^2^). A chin rest was used to prevent head movements. Eye movements were recorded by a desktop-mounted eye tracker (EyeLink 1000 Plus; SR Research, Ottawa, ON, Canada), with a 1000-Hz sampling rate. Participants performed the task binocularly but only the left eye was recorded. A standard 9-point calibration routine was conducted. A standard keyboard and mouse were used to record the participants’ responses.

### Experimental procedure

Two experiments were conducted. In Experiment 1, saccade sensorimotor serial dependencies were investigated; in Experiment 2, visual serial dependencies were investigated. Each experiment included three sessions. In the first session of each experiment, voluntary saccades were performed; in the second, delayed saccades; and, in the third, voluntary and delayed saccades alternated (e.g., trial*_n_*_–__2_ voluntary, trial*_n_*_–__1_ delayed, trial*_n_* voluntary) across trials. The order of sessions within each experiment was fixed, and all subjects performed the three sessions in the described order.

### Saccade reaction time relative to saccade target onset

To ensure comparable visual input and task structure between voluntary and delayed saccade trials, the same visual display (fixation point and peripheral target) was presented in both conditions and remained visible until saccade onset. Because we aimed to match saccade reaction times relative to target onset in the voluntary and delayed saccade sessions, we first measured voluntary saccades. We then presented the acoustic go-signal at a time that would yield reaction times in the delayed saccade trials that were comparable to the voluntary trials. In a previous study, reaction times of saccades triggered by an acoustic go-signal lasted about 150 ms (Zimmermann, 2013). In the previous study, as in the current study, the position of the saccade target was fully predictable, such that saccades likely involved preplanning. Under these circumstances, saccades are known to be initiated faster than usual (Kalesnykas & Hallet, 1987). We presented the acoustic go-signal when subjects had directed their gaze to the fixation point for 100 ms. This procedure ensured that the time interval between saccade target onset and saccade initiation was comparable across both conditions, thus minimizing differences in visual processing duration. Delayed and voluntary saccade sessions had 400 trials each (duration of 20 minutes each). The third session, in which voluntary and delayed saccades alternated, had 800 trials (duration of 40 minutes, beginning with a voluntary saccade trial).

## Experiment 1

### Voluntary saccade session

**Figure 1.**
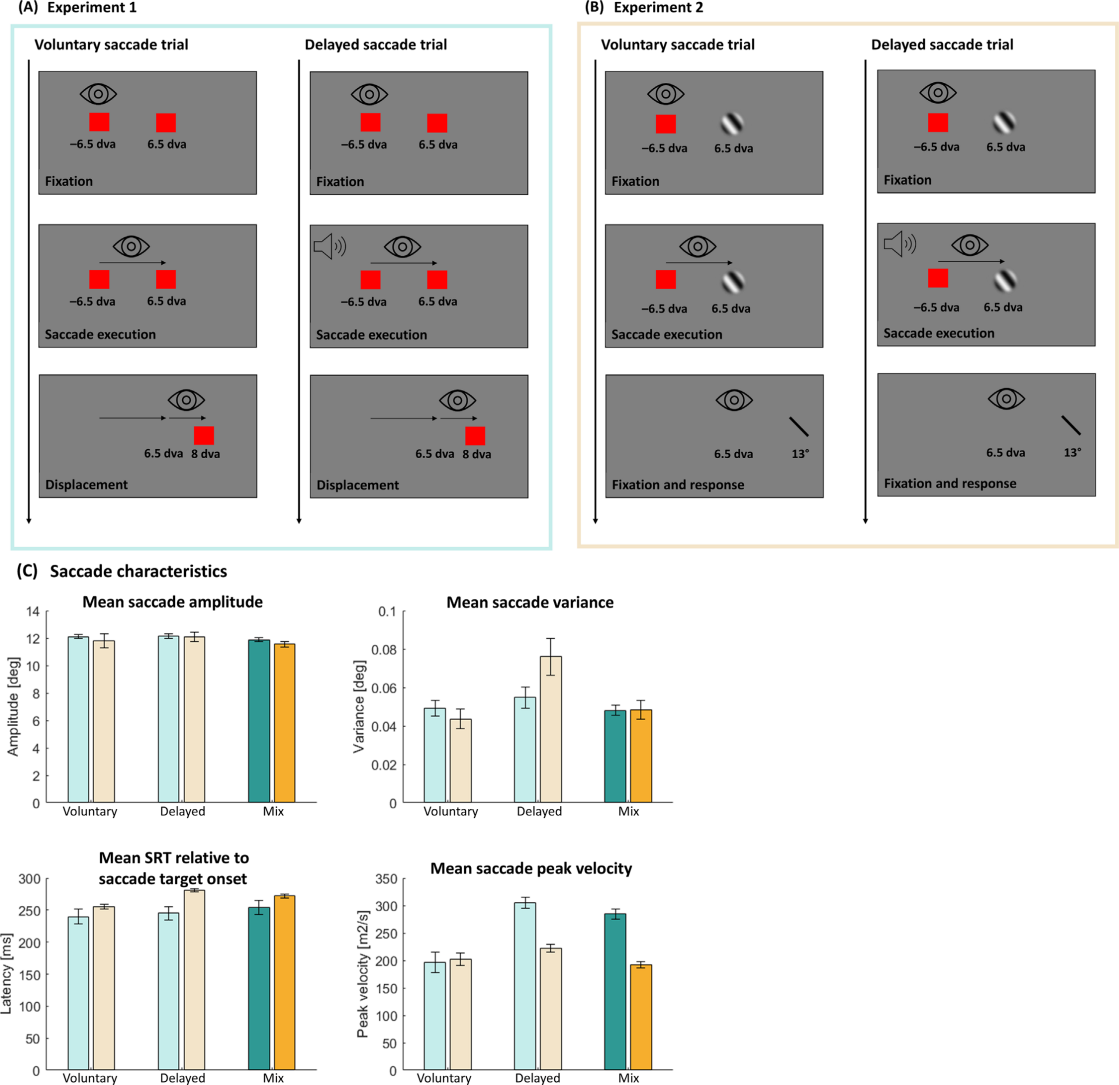
(**A**) Schematic description of the procedure for voluntary and delayed saccade trials (Experiment 1). Subjects performed a saccade toward the target, either voluntarily (left) or delayed (right). During saccade execution, the target was displaced to one out of six possible positions. In delayed saccades trials, the saccade execution was indicated by an acoustical cue. (**B**) Schematic description of the procedure for the orientation judgment task (Experiment 2). The structure was similar to that shown in (**A**), but subjects performed a saccade toward a Gabor patch. After saccade execution (either voluntarily or delayed) subjects maintained fixation and reproduced the perceived orientation by rotating a response bar. (**C**) Average saccade characteristics for saccadic amplitude, variance, SRT relative to saccade target onset, and peak velocity were specified for Experiment 1 (blue) and Experiment 2 (yellow). Lighter colors indicate the condition with the same saccade type; darker colors, the mixed condition. Error bars represent the standard error of the mean.

Figure 1A schematically shows the trial structure. A trial began with the presentation of a fixation square (red, 0.55 degree visual angle [dva] × 0.55 dva) that was shown 6.5 dva to the left side of the screen and the saccade target T1 (red, 0.55 dva × 0.55 dva) that was shown 6.5 dva to the right of the screen center. Subjects were instructed to perform a saccade toward saccade target T1 at their own pace. Eye movements were recorded online, and, as soon as the stimulus program detected an eye velocity higher than 30 dva/s in five consecutive eye-tracking samples, the target was displaced. One target displacement size was randomly selected out of six possible (–2.5, –1.5, –0.5, 0.5, 1.5, or 2.5 dva). The second target disappeared and a new trial started 1200 ms after saccade completion.

### Delayed saccade session

A trial began with the presentation of a fixation square (red, 0.55 dva × 0.55 dva) that was shown 6.5 dva to the left side of screen center and a saccade target T1 (red, 0.55 dva × 0.55 dva) that was shown 6.5 dva to the right side of screen center. Subjects were instructed to perform a saccade as soon as they heard a sinus sound cue. To ensure comparable processing time of the peripheral target across conditions, we measured each subject's mean and standard deviation of saccadic reaction times (SRTs) relative to saccade target onset from the voluntary saccade session and used these values to determine the timing of the auditory go-signal in the delayed saccade trials. The rest of the trial was identical to the voluntary saccade sessions.

## Experiment 2

### Voluntary saccade session

A trial began with the presentation of a fixation square (red, 0.55 dva × 0.55 dva, 6.5 dva to the left of the screen center) and a Gabor patch (T1) that was shown 6.5 dva to the right of the screen center (Figure 1B). The Gabor patch had a spatial frequency of 0.3 cycles per degree and a Gaussian contrast envelope of 1.5° *SD*. The orientation of the Gabor patch randomly varied among five possible orientations (25°, 35°, 45°, 55°, or 65°). Subjects were instructed to perform a voluntary saccade toward T1 and to maintain fixation after saccade landing. As soon as saccade landing was detected (eye velocity smaller than 30 dva/s in five consecutive samples) the Gabor patch disappeared and a response bar (width of 0.80 dva) occurred simultaneously in their periphery. The spatial distance between the saccade landing position and the response bar location was adjusted to correspond with the size of the saccade amplitude. This adjustment ensured that T1 (while fixating on the left) and the response bar (while fixating on T1 location) maintained the same retinal position. The orientation of the response bar was randomly determined for each trial. Participants were instructed to align the orientation of the response bar with their perceived orientation of the Gabor patch. Using a standard computer mouse, participants could rotate the response bar either clockwise or counterclockwise. They confirmed their response by pressing the space bar.

### Delayed saccade session

Delayed saccades trials were designed as in the delayed saccades session of Experiment 1. The fixation and Gabor patch were presented simultaneously and remained visible until saccade onset. Instead of red target squares, Gabor patches were used as targets with the same characteristics and task as described for the voluntary saccade trials for Experiment 2.

### Data analyses

All saccades with amplitudes larger than half the required distance were included in the analysis. In delayed saccade trials, we excluded trials in which subjects performed anticipatory saccades, which started before the sinus sound cue was played. For Experiment 2, we additionally excluded trials in which participants did not fixate the Gabor patch location after saccade execution—that is, in which gaze positions exceeded a radius of 2.5 dva around the Gabor patch. On average, ∼95% of trials went into analysis.

For Experiment 1, we computed the postsaccadic error for each trial as the difference between the saccade landing and the position of the displaced target in this trial. For Experiment 2, we computed the deviation error between the Gabor patch orientation and the reproduced orientation. In order to analyze serial dependencies in Experiment 1, we calculated linear regressions between the postsaccadic error in the previous trial and the landing error relative to T1 in the current trial for each subject in each session. In Experiment 2, we calculated linear regressions between the stimulus orientation in the previous trial and the deviation error between the stimulus orientation and the reproduced orientation in the current trial for each subject in each session. Bonferroni-corrected Student's *t*-tests against zero on the slopes were conducted to investigate serial dependence effects. We conducted a 2 × 2 analysis of variance (ANOVA) with the within-subject factor “previous trial” (voluntary, delayed) and the within-subject factor “current trial” (voluntary, delayed) to investigate differences in the strength of trial-by-trial influences.

## Results

### Experiment 1

**Figure 2.**
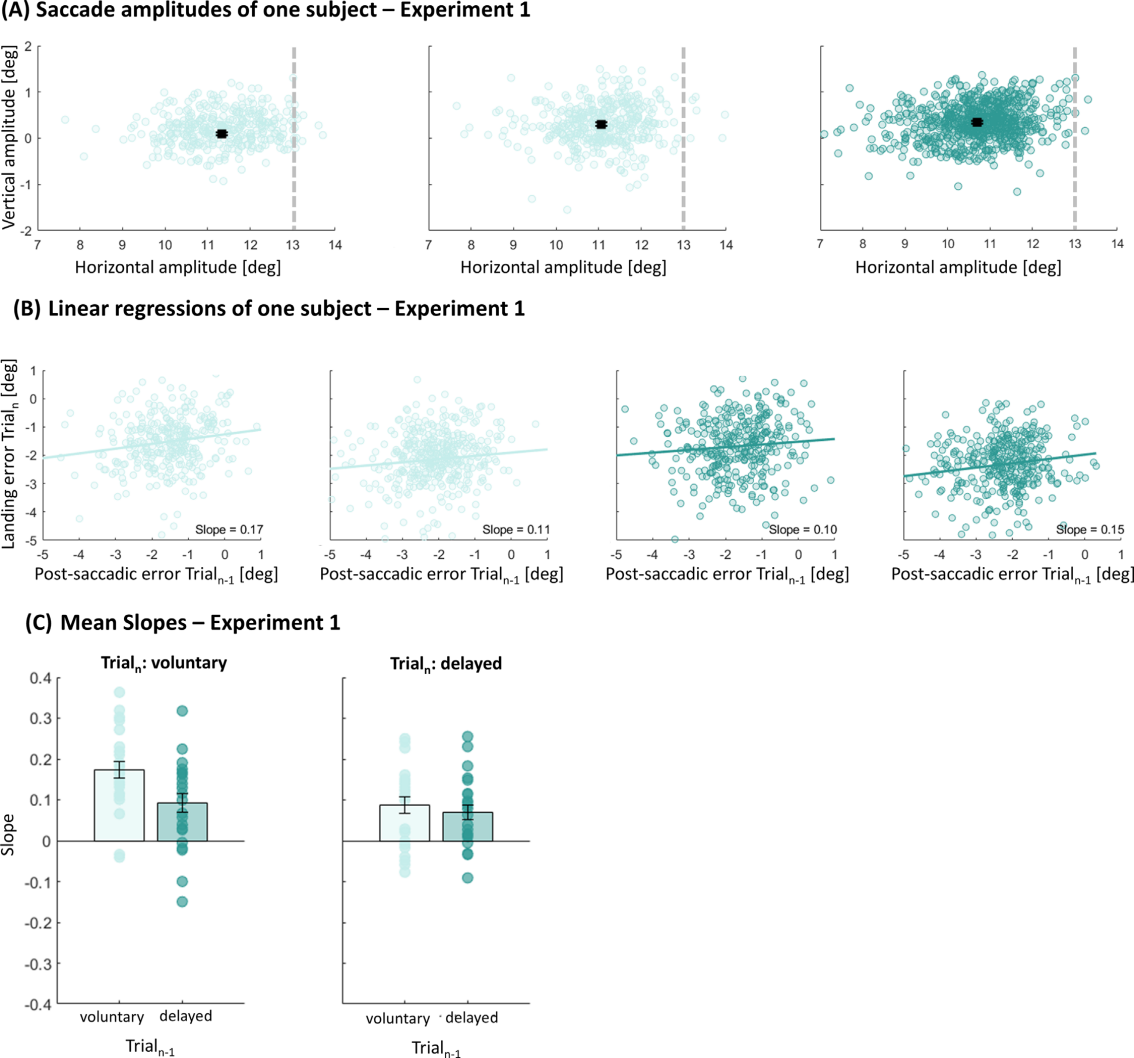
(**A**) Saccadic amplitudes of one example subject for each session in Experiment 1. The dashed line represents the optimal amplitude to reach the target. The black square represents the mean saccadic amplitude. Error bars represent standard error of the mean. (**B**) Presentation of the landing error relative to T1 in trial*_n_* as a function of the postsaccadic error of trial*_n_*_–1_ of one example subject for each session. Positive errors were interpreted as saccades overshooting the target and negative numbers represent a saccadic undershoot. The positive slope (solid line) reveals that larger postsaccadic errors in the previous trial led to larger landing errors relative to T1 in the current trial. (**C**) Mean slopes for the linear regression between the predictor “previous trial” (voluntary or delayed saccades) and the within-subject factor “current trial” (voluntary or delayed saccades). Only when a voluntary saccade was performed was the last behavior taken into consideration if it was a delayed saccade. Error bars represent the standard error of the means.

Figure 2A shows saccadic amplitudes for a representative subject in Experiment 1. Subjects were instructed to perform a horizontal saccade of 13° (indicated by the dashed line in all panels). In all sessions, the subject undershot the target systematically. In the left panel, saccadic amplitudes for only voluntary saccades are presented, resulting in a mean saccadic amplitude of 11.34 dva (*SD* = 0.03 dva). In the middle panel, only delayed saccades were performed (mean amplitude, 11.06 ± 0.03 dva). In the right panel, the subject performed voluntary and delayed saccades alternatingly, starting with voluntary saccades (mean amplitude, 10.70 ± 0.03 dva).

We calculated the postsaccadic error between the actual target position and the saccadic amplitude. In Figure 2B, one example subject for each session is presented to visualize the magnitude of saccade-by-saccade influences. Negative numbers indicate an undershooting of saccadic amplitude, and positive numbers indicate an overshooting of the target. To investigate serial dependencies of the postsaccadic error from the previous trial (trial*_n_*_–1_) to the landing error relative to T1 in the current trial (trial*_n_*), we fitted a linear regression model for each subject in every session separately. In this experiment, horizontal saccades were performed: only voluntary (first panel), only delayed (second panel), voluntary and delayed (third panel), or delayed and voluntary (fourth panel) horizontal saccades. We used the slopes of the linear fits to quantify the magnitude of sensorimotor serial dependencies. Positive slopes indicate a positive serial dependency between postsaccadic errors: Larger postsaccadic errors in the trial*_n_* led to larger postsaccadic errors in trial*_n_*_–1_.

Overall, we found serial dependencies for horizontal saccades, independently of the performed saccade type combination, as Bonferroni corrected *t*-tests against zero for the mean slopes indicate—trial_*n*–1_: voluntary, trial_*n*_: voluntary, *t*(22) = 8.63, *p* < 0.001; trial_*n*–1_: delayed, trial_*n*_: delayed, *t*(22) = 4.41, *p* < 0.001; trial_*n*–1_: voluntary, trial_*n*_: delayed, *t*(22) = 3.86, *p* < 0.001; trial_*n*–1_: delayed, trial_*n*_: voluntary, *t*(22) = 4.10, *p* < 0.001 (Figure 2C).

Additionally, we were interested if the serial dependence strength differs if we currently perceive a voluntary or delayed saccade trial, dependent on the previously performed saccade trial (voluntary, delayed). A 2 × 2 ANOVA with the factor “previous trial” (voluntary, delayed) and the within-subject factor “current trial” (voluntary, delayed) for horizontal saccades indicated a significant main effect of “previous trial,” *F*(1,22) = 4.75, *p* = 0.040, and “current trial,” *F*(1,22) = 9.46, *p* = 0.006, in the serial dependence strength, as well a significant interaction effect, *F*(1,22) = 4.42, *p* = 0.047. Bonferroni-corrected post hoc tests indicated stronger serial dependencies for trials in which only voluntary saccades were performed compared with only delayed saccades (*t* = 3.76, *p* = 0.003), voluntary saccades followed by delayed saccades (*t* = 3.57, *p* = 0.006), and delayed saccades followed by voluntary saccades (*t* = 2.95, *p* = 0.033). In contrast, when subjects performed a delayed saccade, the previously performed postsaccadic error did not lead to significantly different serial dependence strengths compared with all other sessions.

For the experimental design, we determined when the acoustic go-signal had to be presented to obtain SRTs relative to target onset that were comparable between voluntary and delayed saccades. An independent *t*-test of the SRTs did not reveal any significant difference, *t*(44) = 0.12, *p* = 0.902. There is thus no evidence to assume that reaction times, and thereby the duration of saccade target visibility, differed between saccade types.

**Figure 3.**
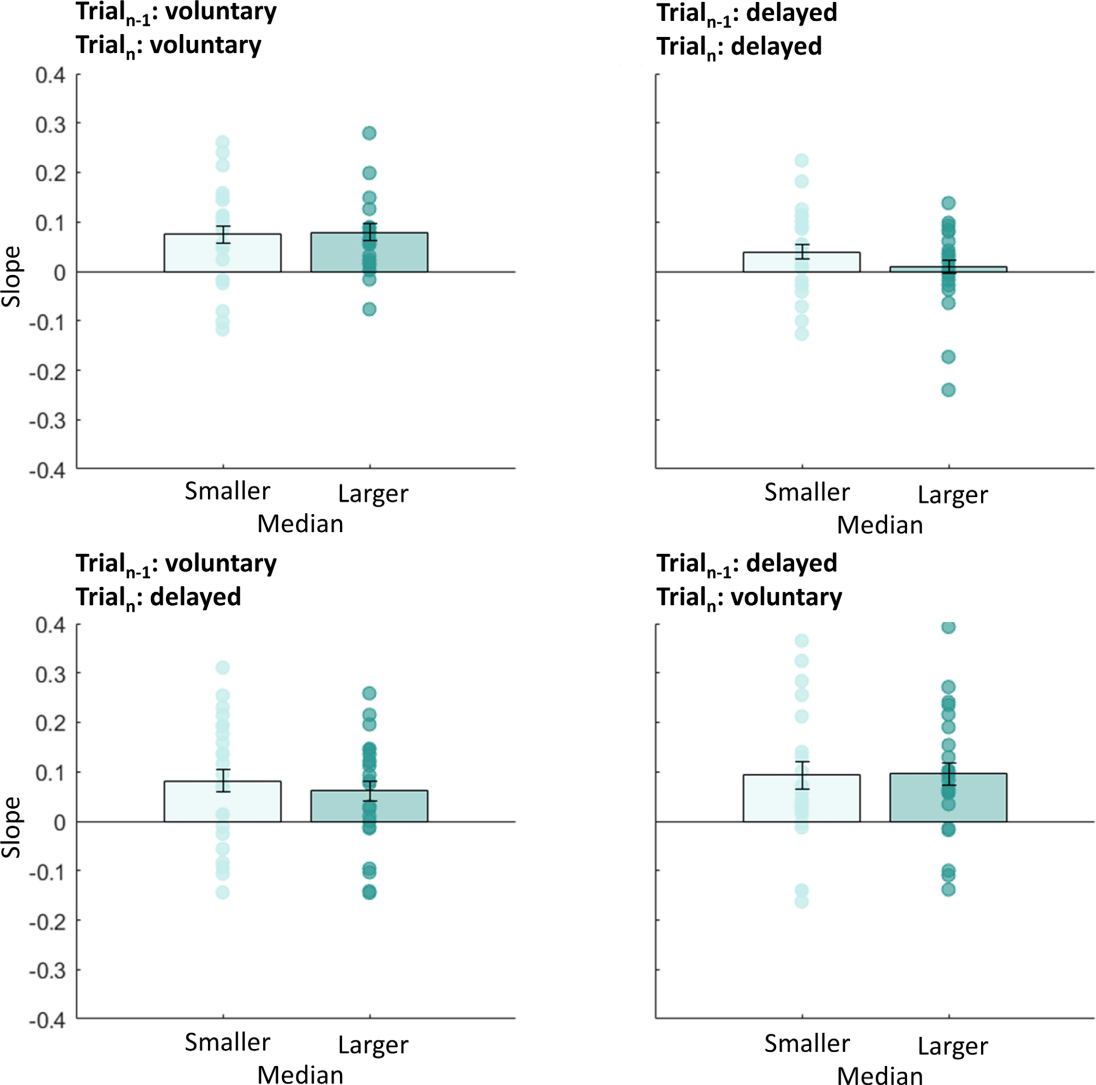
Mean slopes for the linear regression between the predictor “previous trial” (voluntary or delayed) and the within-subject factor “current trial” (voluntary or delayed), separated for trials with a saccadic amplitude smaller (light blue) or larger (dark blue) than the median saccade amplitude for Experiment 1. Error bars represent the standard error of the means.

**Figure 4.**
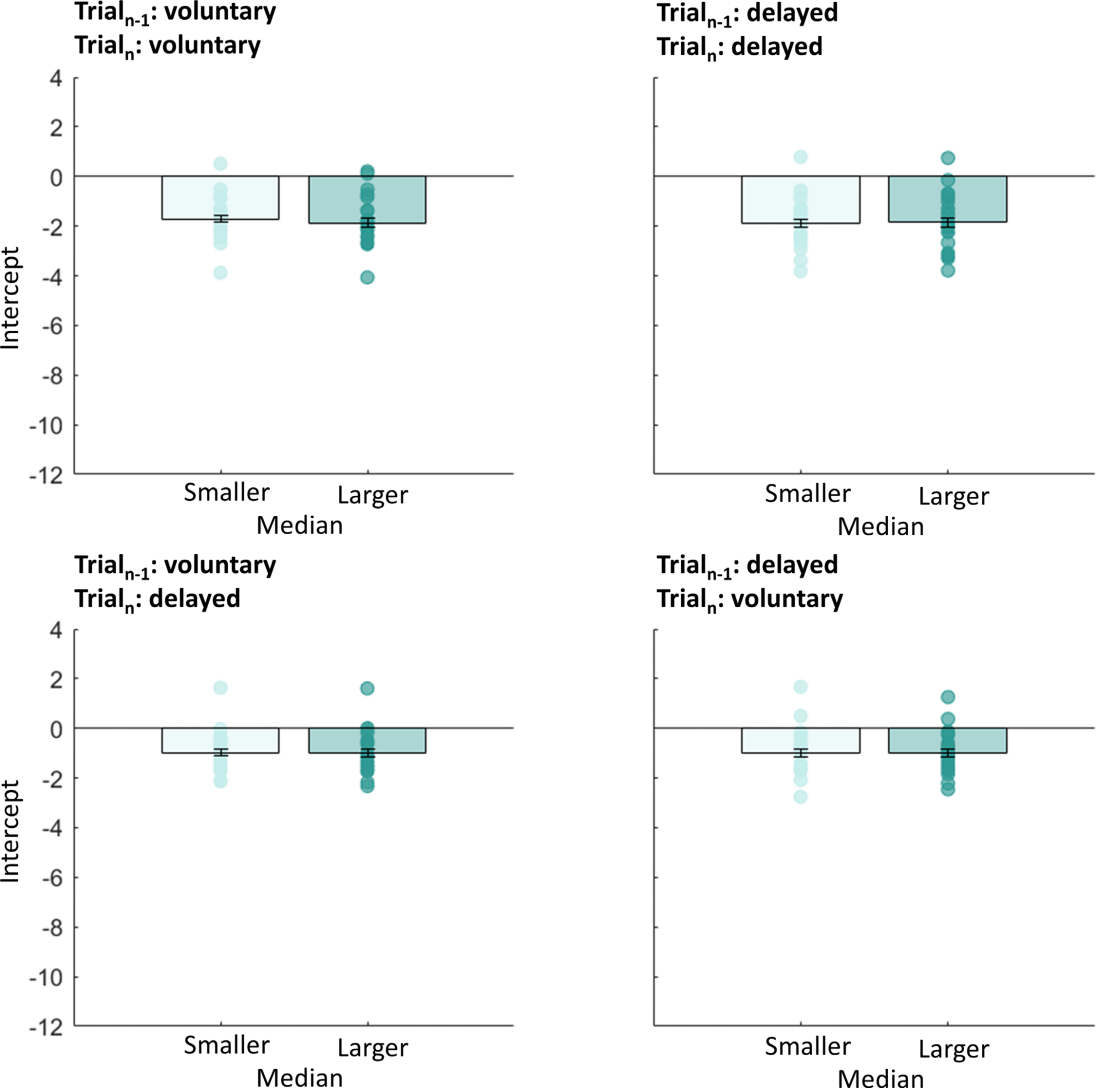
Mean intercepts for the linear regression between the predictor “previous trial” (voluntary or delayed) and the within-subject factor “current trial” (voluntary or delayed), separated for trials with a saccadic amplitude smaller (light yellow) or larger (dark blue) than the median saccade amplitude for Experiment 1. Error bars represent the standard error of the means.

Most saccades undershot target T1. We wondered whether this mistargeting was predicted by the sensorimotor system. If these errors in landing were not predicted, then saccades with higher undershoot would produce larger postsaccadic retina errors. In that case, we would expect stronger serial dependencies for saccades with larger saccade undershoot. We split all data into two groups in which we selected saccades with amplitudes lower or higher than the median (mdn) saccade amplitude (*mdn*_volu__n__tary_ = 12.22 dva, *mdn*_delayed_ = 12.19 dva, *mdn*_volu__n__tary-delayed_ = 11.94 dva, *mdn*_delayed-volu__n__tary_ = 11.94 dva). Independent *t*-tests between the two groups for each saccade type (voluntary, delayed, voluntary-delayed, or delayed-voluntary) indicated no significant difference in slopes—trial*_n_*_–1_: voluntary, trial*_n_*: voluntary, *t*(44) = 0.14, *p* = 0.889; trial*_n_*_–1_: delayed, trial*_n_*: delayed, *t*(44) = 1.26, *p* = 0.214; trial*_n_*_–1_: voluntary, trial*_n_*: delayed, *t*(44) = 0.06, *p* = 0.951; trial*_n_*_–1_: delayed, trial*_n_*: voluntary: *t*(44) = 0.12, *p* = 0.586 (see also Figure 3). We performed the same analysis for the intercepts of the fits (see Figure 4) to investigate the deviation error in the current trial, but found no influence of the previous trial. Independent *t*-tests on the intercepts indicated no significant difference—trial*_n_*_–1_: voluntary, trial*_n_*: voluntary, *t*(44) = 0.60, *p* = 0.551; trial*_n_*_–1_: delayed, trial*_n_*: delayed, *t*(44) = 0.07, *p* = 0.947; trial*_n_*_–1_: voluntary, trial*_n_*: delayed, *t*(44) = 0.01, *p* = 0.988; trial*_n_*_–1_: delayed, trial*_n_*: voluntary: *t*(44) = 0.04, *p* = 0.961 (see also Figure 4).

### Experiment 2

**Figure 5.**
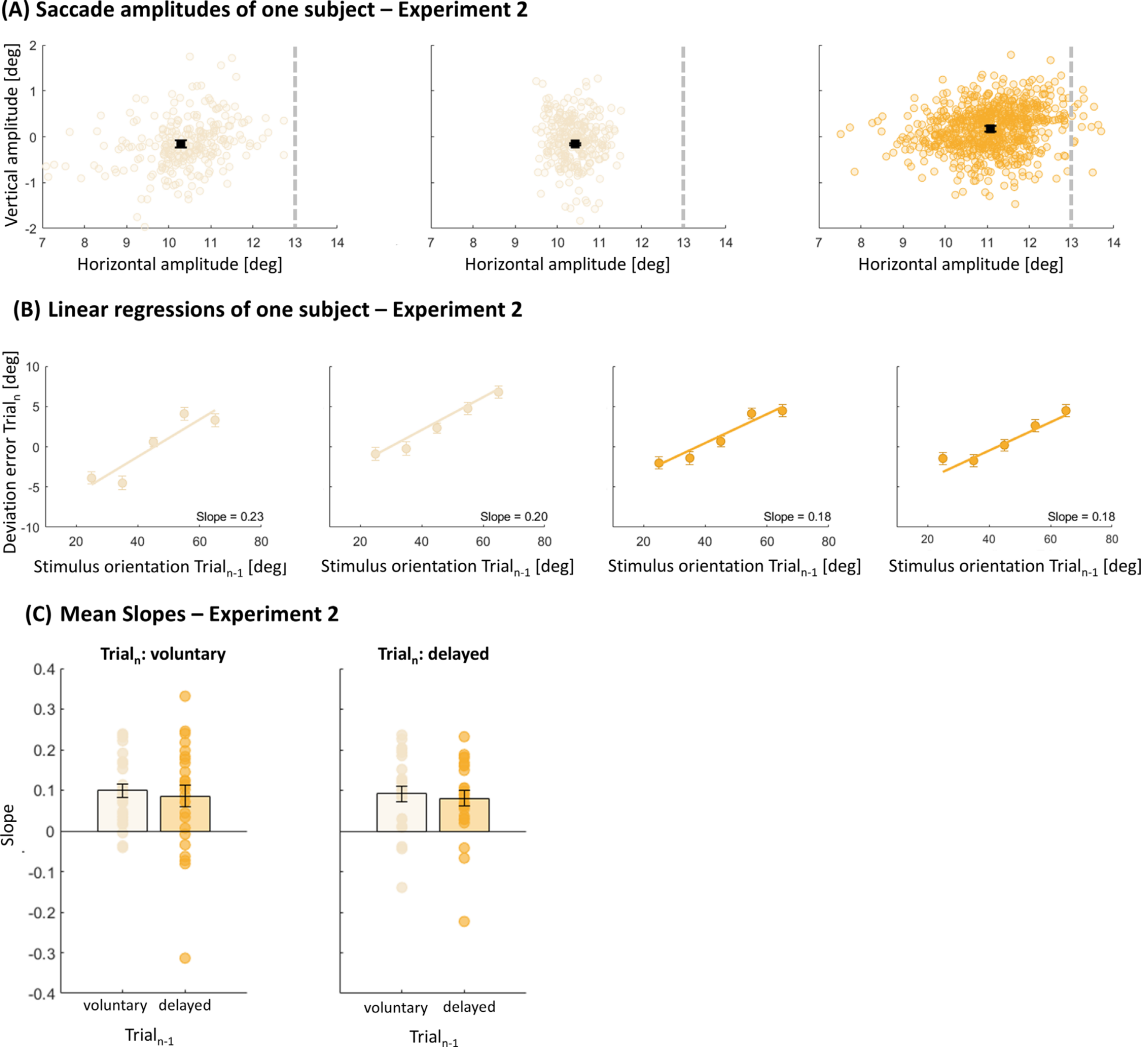
(**A**) Saccadic amplitudes of one example subject for each session. The dashed line represents the center of the saccade target (i.e., the Gabor patch). The black square represents the mean saccadic amplitude. Error bars represent standard error of the mean. (**B**) Presentation of the deviation error in trial*_n_* as a function of target orientation in trial*_n_*_–1_ of one example subject for each session. Positive errors are interpreted as overestimations and negative numbers represent underestimations. The positive slope (solid line) reveals that larger orientations in the previous trial led to larger deviation errors in the current trial. (**C**) Mean slopes for the linear regression between the predictor “previous trial” (voluntary or delayed saccades) and the within-subject factor “current trial” (voluntary or delayed saccades) for both experiments. No differences in the serial dependency magnitude were found. Error bars represent the standard error of the means.

Figure 5A shows saccadic amplitudes for a representative subject in Experiment 2. Subjects were instructed to perform a horizontal saccade of 13 dva (indicated by the dashed line in all panels) before judging the orientation of the target stimulus. In the left panel, saccadic amplitudes for only voluntary saccades are presented, resulting in a mean saccadic amplitude of 10.29 dva (*SD* = 0.08 dva). In the middle panel, only delayed saccades were performed (mean amplitude, 10.42 ± 0.02 dva). In the right panel, the subject performed voluntary and delayed saccades alternatingly, starting with voluntary saccades (mean amplitude, 11.07 ± 0.06 dva).

We calculated the deviation error between the physically presented and the reproduced orientation. In Figure 3B, one example subject for each session is presented to visualize the magnitude of saccade-by-saccade influences for Experiment 2. To investigate serial dependencies of the presented orientation of the previous trial (trial*_n_*_–1_) to the deviation error of the current trial (trial*_n_*), we fitted a linear regression model for each subject in every session separately. Orientation judgments were required after either only voluntary saccades (first panel), only delayed saccades (second panel), voluntary and delayed saccades (third panel), or delayed and voluntary saccades (fourth panel).

We found serial dependence influences for all four sessions, independently of the performed saccade type combination, as *t*-tests against zero for the mean slopes indicated—trial*_n_*_–1_: voluntary, trial*_n_*: voluntary, *t*(22) = 6.03, *p* < 0.001; trial*_n_*_–1_: delayed, trial*_n_*: delayed, *t*(22) = 5.13, *p* < 0.001; trial*_n_*_–1_: voluntary, trial*_n_*: delayed, *t*(22) = 4.35, *p* < 0.001; trial*_n_*_–1_: delayed, trial*_n_*: voluntary, *t*(22) = 3.28, *p* = 0.003 (Figure 5C).

A 2 × 2 ANOVA with the factor “previous trial” (voluntary, delayed) and the within-subject factor “current trial” (voluntary, delayed) indicated no differences in serial dependency strengths: main effect previous trial, *F*(1,22) = 0.11, *p* = 0.917; main effect current trial, *F*(1,22) = 0.17, *p* = 0.608; interaction effect previous trial × current trial, *F*(1,22) = 0.47, *p* = 0.498. Independently of the performed saccade type in the current and previous trial, orientation judgment errors influenced equally strongly from trial to trial.

**Figure 6.**
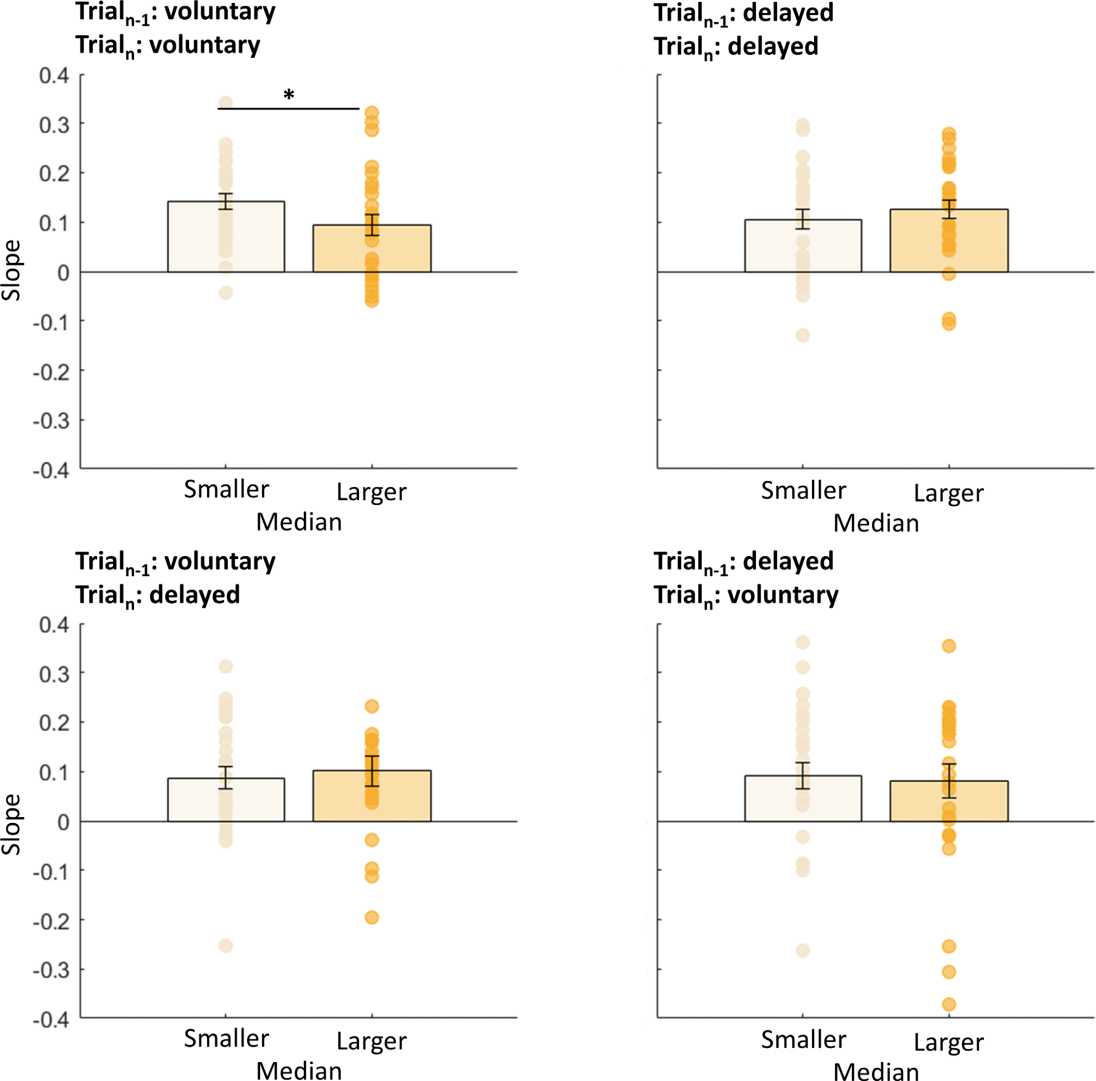
Mean slopes for the linear regression between the predictor “previous trial” (voluntary or delayed) and the within-subject factor “current trial” (voluntary or delayed), separated for trials with a saccadic amplitude smaller (light yellow) or larger (dark yellow) than the median saccade amplitude for Experiment 2. We found a significant difference only in the session in which subjects were instructed to perform only voluntary saccades. Error bars represent the standard error of the means.

To further explore the absence of results in Experiment 2, we conducted a median split. As for Experiment 1, we split the data into groups with saccades smaller or larger than the median saccade amplitude (*mdn*_volu__n__tary_ = 12.02 dva, *mdn*_delayed_ = 10.65 dva, *mdn*_volu__n__tary-delayed_ = 11.21 dva, *mdn*_delayed-volu__n__tary_ = 11.21 dva). Independent *t*-tests between the two groups for each saccade type (voluntary, delayed, voluntary-delayed, or delayed-voluntary) indicated only a significant difference in slopes of voluntary saccades, with more clearly higher trial-by-trial influences for undershooting saccades compared with overshooting saccades, *t*(44) = 2.06, *p* = 0.050—trial*_n_*_–1_: delayed, trial*_n_*: delayed, *t*(44) = 1.02, *p* = 0.318; trial*_n_*_–1_: voluntary, trial*_n_*: delayed, *t*(44) = 0.48, *p* = 0.635; trial*_n_*_–1_: delayed, trial*_n_*: voluntary, *t*(44) = 0.37, *p* = 0.717 (see also Figure 6).

**Figure 7.**
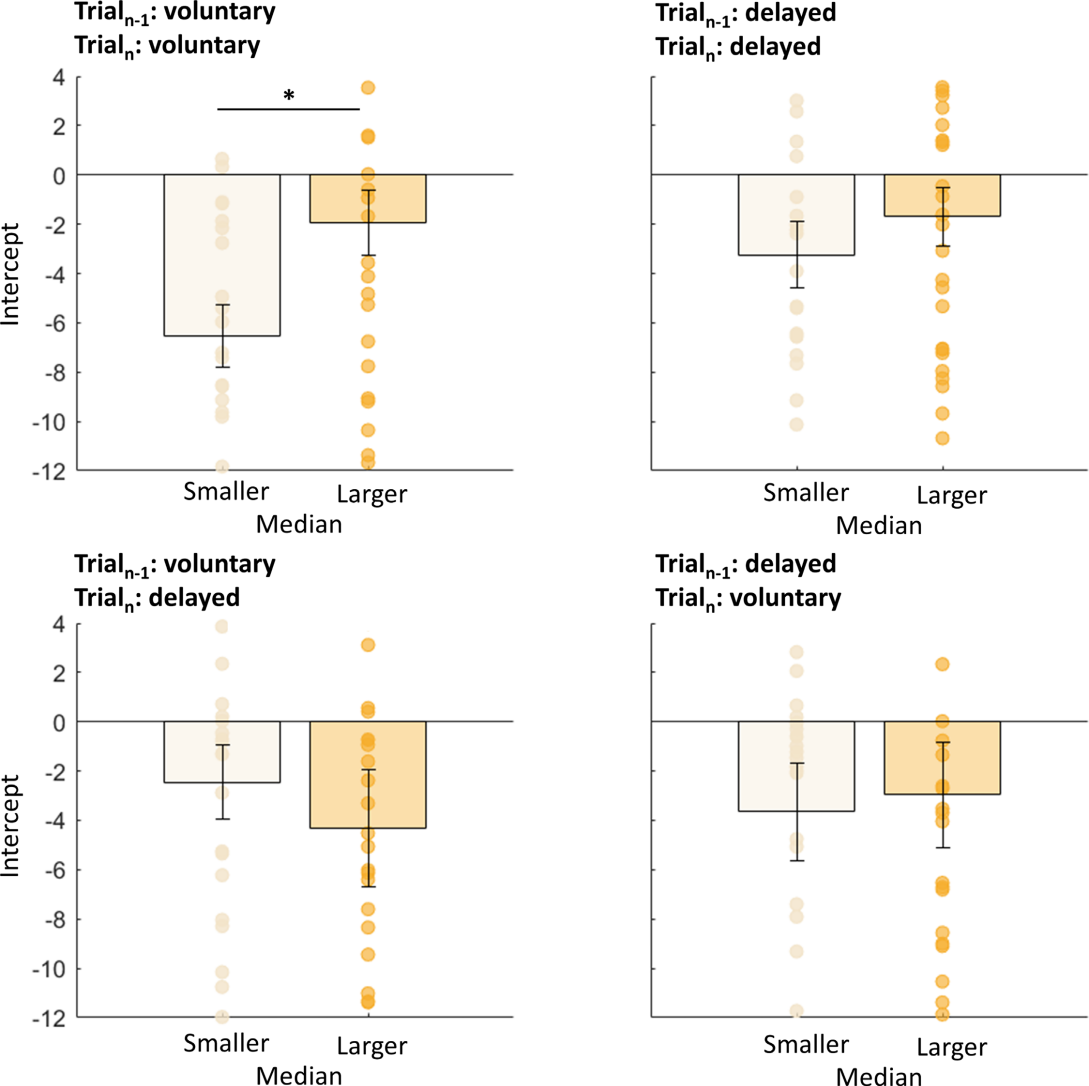
Mean intercepts for the linear regression between the predictor “previous trial” (voluntary or delayed) and the within-subject factor “current trial” (voluntary or delayed), separated for trials with a saccadic amplitude smaller (light yellow) or larger (dark yellow) than the median saccade amplitude for Experiment 2. We found a significant difference only in the session in which subjects were instructed to perform only voluntary saccades. Error bars represent the standard error of the means.

We performed the same analysis for the intercepts of the fits (see Figure 7) to investigate the deviation error in the current trial but found no influence of the previous trial. Independent *t*-tests on the intercepts indicated only a significant difference for the voluntary saccade type, *t*(44) = 4.85, *p* < 0.001—trial*_n_*_–1_: delayed, trial*_n_*: delayed, *t*(44) = 1.51, *p* = 0.143; trial*_n_*_–1_: voluntary, trial*_n_*: delayed, *t*(44) = 1.17, *p* = 0.254; trial*_n_*_–1_: delayed, trial*_n_*: voluntary, *t*(44) = 0.61, *p* = 0.549 (see also Figure 7). We found higher trial-by-trial influences for undershooting saccades compared with overshooting saccades.

## Discussion

In this study, we compared the influence of delayed and voluntary saccade performance on serial dependencies in saccade landings and in visual orientation judgments. We found that serial dependencies in saccade landings were selective for the saccade type. If voluntary saccades were performed in the previous and in the current trial, the magnitude of amplitude adjustments was significantly higher than if voluntary saccades were followed by delayed saccades. However, if delayed saccades were performed in the previous trial, amplitude adjustments were equally strong irrespective of whether delayed or voluntary saccades were executed in the following trial.

We reasoned that the asymmetric transfer could be related either to the programming of saccades (voluntary/delayed) or to the way visual attention is drawn to the saccade target (endogenous/exogenous). In the latter case, asymmetric transfer should also be observable for visual features if attended to either endogenously or exogenously. To this end, we also measured serial dependency strength on visual orientation judgments for oriented targets toward which subjects performed either delayed or voluntary saccades. Serial dependencies were equally strong, irrespective of the saccade types that had been performed toward the targets. In summary, in our study, we found that serial dependencies in saccade amplitude shifts are selective for the saccade type, but serial dependencies in orientation judgments are independent of it.

The asymmetric transfer of serial dependencies in motor errors and amplitude adjustments between delayed and voluntary saccades might reflect the different programming stages for both saccade types (Deubel, 1995). If the postsaccadic error was processed differently due to the different covert attentional deployment, one would expect stronger serial dependencies in one of the two saccade types. However, serial dependencies were equally strong between delayed and voluntary saccades in blocks in which only one saccade type was performed.

Our data replicate the asymmetric transfer of motor error information that has been reported previously in the saccade adaptation literature (Alahyane et al., 2007; Collins & Doré-Mazars, 2006; Deubel, 1995; Erkelens & Hulleman, 1993). Our results are consistent with the idea that differences in motor error transfer between voluntary and delayed saccades are found at the saccade programming stages. In an early model, it was suggested that voluntary saccade adaptation might reside in frontal areas and delayed saccade adaptation in the superior colliculus (Deubel, 1995). Given that the frontal areas are higher up in the hierarchy, the asymmetric transfer of adaptation would be explainable. If adaptation occurred in the frontal areas, delayed saccades, being programmed more downstream, would remain uninformed of it. In the opposite case, voluntary saccades would be affected by adaptation in the colliculus, through which voluntary saccade planning signals would pass. However, neural activation corresponding to saccade adaptation does not support such an easy picture.

Electrophysiological studies (Gerardin, Miquée, Urquizar, & Pélisson, 2012; Guillaume, Fuller, Srimal, & Curtis, 2018; Métais et al., 2022) and positron emission tomography studies (Desmurget et al., 2000; Desmurget et al., 1998) have highlighted the pivotal importance of the cerebellum for saccade adaptation. The cerebellum detects and processes the postsaccadic error (Herzfeld, Kojima, Soetedjo, & Shadmehr, 2018) and might also be responsible for amplitude adjustments. However, two patient studies found that lesions in thalamic nuclei that transport information from the cerebellum to cerebellar areas diminish saccade adaptation magnitude. Functional brain imaging in humans was used to investigate neural activation corresponding to delayed and voluntary saccades (Corbetta & Shulman, 2002). The study found activation in middle-temporal, temporoparietal, and frontal areas for delayed saccade adaptation. Voluntary saccade adaptation included the same areas and in addition parietal areas. The authors argued that this dissociation matches the dorsal/ventral specialization of parietofrontal streams relative to covert shifts of visual attention (Corbetta & Shulman, 2002). Guillaume et al. (2018) suggested that neural activation in the classical saccade adaptation paradigm might result either from adaptation or from saccade error processing. Because usually an adaptation task is compared to a control task in which no intrasaccadic target displacement is applied, cortical activation might reflect the error but not the adaptation. By displacing the target only during saccade execution and then clamping the target close to saccade landing, Guillaume et al. (2018) avoided that confound; under this condition, they found activation of parietal and frontal areas involved in the adaptation of delayed saccades. The involvement of frontal areas in delayed saccade adaptation that was not attributable to mere error processing was also confirmed in a functional magnetic resonance imaging localizer study (Métais et al., 2022).

In visual orientation judgments, serial dependencies were equally strong irrespective of whether delayed saccades, voluntary saccades, or alternating delayed and voluntary saccades were performed. The interpretation of motor-type specificity is in line with the absence of any difference between delayed and voluntary saccades on sensory serial dependencies. Serial dependencies in vision have been argued to stabilize perception by smoothing sensory input toward previous experiences. Because serial dependencies are concerned with the interpretation of the external world, they should generalize about whether objects were attended endogenously or exogenously. It is unclear whether results observed in experiments involving covert attention shifts can be used to interpret findings from paradigms involving overt attention shifts. There is no guarantee that overt and covert attention shifts are always coupled. In contrast, Casteau and Smith (2020) provided evidence that only exogenous overt attention is coupled to eye movement programming. Endogenous overt attention, however, appeared to be independent of it as it could be directed to regions of the visual field that would be unreachable with eye movements. The independence of endogenous overt attention from eye movement programming might also explain the asymmetric transfer of adaptation.

In conclusion, the current study demonstrated that exogenous and endogenous attention shifts differentially affect sensorimotor serial dependencies but not visual serial dependencies.
